# “I Really Dreamed of True Love”: An Ethnography on Gender‐Based Violence and Its Effects on the Healthcare of Women Experiencing Homelessness

**DOI:** 10.1111/phn.70182

**Published:** 2026-09-21

**Authors:** Julia Marino Sanches, Vitor Rafael Gomes, Bárbara Martins, Disete Devera, Luciane Miranda Guerra, Luiz Francesquini Júnior

**Affiliations:** ^1^ State University of Campinas (UNICAMP) Campinas São Paulo Brazil; ^2^ Fiocruz Brasília Brasília, Distrito Federal Brazil

**Keywords:** gender‐based violence, homeless population, women's health

## Abstract

**Objective:**

To understand how affective bonds and experiences of gender‐based violence intersect the trajectories of women experiencing homelessness and influence their relationship with the Psychosocial Care Network (RAPS).

**Methods:**

A qualitative study with an ethnographic approach, conducted between August and December 2025 in Campinas, Brazil, alongside “Street Clinic” (Consultório na Rua) teams. Participants were 11 adult women experiencing homelessness. Data were produced through participant observation, semi‐structured interviews, and field diaries, and subjected to Thematic Content Analysis.

**Results:**

The study identified the centrality of intimate partner and institutional violence in the participants’ trajectories and their impacts on care. It highlighted the ambivalence of heterosexual affective‐romantic bonds, which operate paradoxically as sources of protection, belonging, and emotional support, but also as spaces for reproducing inequalities, restricting autonomy, and creating barriers to healthcare access and continuity.

**Conclusions:**

Romantic bonds occupy a central position in these women’s daily lives, placing them between protection and vulnerability. Care experiences are intersected by multiple social markers, reinforcing the need for public policies guided by intersectional, gender‐sensitive perspectives capable of recognizing the complexity of care itineraries for this population.

## 1 Introduction

The homeless population (PSR) constitutes one of the social groups most exposed to processes of vulnerability, marked by precarious living conditions, weakened social and family ties, food insecurity, and a high burden of illness (Brazil 2009). Access to health services presents itself as a daily challenge, permeated by institutional, symbolic, and material barriers (Brazil 2009; Silva 2024).

Although the Brazilian Unified Health System (SUS) is guided by the principles of universality, comprehensiveness, and equity, the homeless population frequently encounters difficulties accessing health services, whether due to documentation requirements, rigid institutional procedures, discontinuity of care, or experiences of stigmatization, discrimination, and institutional violence (Silva 2024). The Psychosocial Care Network (RAPS) plays a central role in organizing care, relying on mechanisms such as the Street Clinic, considered strategic for expanding access to health services for this population (Brazil 2011).

It is important to highlight that the PSR is predominantly composed of Black people (Obpoprua, 2024). This reality is related to historical processes of inequality produced by colonialism and the enslavement of the Black population, whose effects remain present in the unequal distribution of opportunities, rights, and living conditions (Almeida 2019).

Furthermore, it is a predominantly male population (Obpoprua, 2024). Even so, the existence and specificities of women's trajectories should not be disregarded, so that the analysis of the experiences of women experiencing homelessness demands an intersectional perspective, considering that housing conditions, gender, and race are articulated in the production of specific experiences of vulnerability and violence, as discussed by Gonzalez (2020).

In the lives of these women, violence constitutes an important social marker. Data from the Notifiable Diseases Information System (Sinan) from 2022 reveal that, although they represent only 13% of this population, women account for 40% of violence reports (Brazil 2023).

Furthermore, violence is often one of the triggering events for people to end up on the streets. However, the experience of living without formal housing does not interrupt this cycle: violations continue and diversify, being committed by both institutions and other individuals, including intimate partners. In this context, affective bonds assume a relevant role in their trajectories, and can constitute both strategies for protection and survival, as well as contexts for the reproduction of inequalities and violence (Richwin; Zanello 2023).

It is worth noting that much of the research on homelessness focuses on epidemiological indicators, service use, or sociodemographic characterization, contributing to the visibility of the problem, but sometimes limiting the understanding of the experiences lived by the subjects themselves (Oliveira et al. 2020; Jesus and Lisboa, 2022). The perspective proposed by Viveiros de Castro (2002), by emphasizing the understanding of social phenomena from the points of view of the subjects themselves, recognizing the legitimacy of their ways of knowing and experiencing reality, reinforces the relevance of qualitative studies, especially those of ethnographic inspiration, to understand how access to health is perceived, lived, and negotiated in everyday life, as well as the meanings attributed to care practices, institutions, and relationships established with health professionals.

Although the literature points to the centrality of gender‐based violence in the trajectories of these women (Giacomini; Albuquerque 2021; Richwin; Zanello 2023) and how this severely impacts their health conditions and psychological suffering (Buriola et al., 2018), studies investigating how these relational dynamics concretely affect their relationship with health services are still scarce. It is therefore essential to problematize to what extent relationships with intimate partners affect access to and permanence in the care services of the Psychosocial Care Network (RAPS).

Unlike previous studies that document gender‐based violence as a structural marker of homelessness (Richwin; Zanello 2023) or its effects on the mental health of this population (Buriola et al., 2018), this study seeks to contribute by ethnographically situating how affective bonds, and not just isolated episodes of violence, operate as concrete mediators of access, adherence, and continuity of care in the services of the Psychosocial Care Network. This fills a gap in the processual and situated understanding of how gender‐based violence, perpetrated by both intimate partners and institutions, translates into concrete barriers and possibilities within the therapeutic itineraries of these women. In this context, the present study aims to understand how affective bonds and experiences of gender‐based violence traverse the trajectory of homeless women in accessing services within the Psychosocial Care Network. The goal is to foster care strategies that recognize the uniqueness of these experiences.

## 2 Method

### 2.1 Study Design

This is a qualitative research study with an ethnographic approach, a method that allowed the researcher to immerse herself in the daily lives of the participants, enabling an understanding of the relational dynamics present in the territory and the meanings attributed to care based on lived experiences. The choice of ethnography proved pertinent as it facilitates the understanding of these phenomena within their social and cultural contexts of production (Geertz 1989).

### 2.2 Location and Period of the Research

The study was conducted in the municipality of Campinas (SP), together with the Street Clinic teams, between August and December 2025. The fieldwork focused on locations where the service operates: Largo do Pará, Praça do Teatro Castro Mendes, and in front of Casa da Cidadania, a social assistance facility that provides services to people experiencing homelessness.

### 2.3 Participants and Inclusion Procedures

Eleven adult women experiencing homelessness, users of the Street Clinic, who met the previously established inclusion criteria for the research, participated in the study. The participants were identified during the team's work in the area and invited to participate in the study after expressing interest and availability. Following this initial contact, the researcher extended an individual invitation, presenting the research objectives and the ethical aspects involved.

The inclusion criteria considered were: a) being female; b) being 18 years of age or older; c) being homeless in the municipality of Campinas (SP); d) being a user of the Street Clinic; and e) having at least three months of experience living on the streets.

The following exclusion criteria were established: a) being under 18 years of age; b) not being a user of the Street Clinic; c) having been homeless for less than three months; or d) not being able to participate in the interview and provide free and informed consent at the time of the approach.

### 2.4 Researcher's Position

The construction of this research is inseparable from the researcher's trajectory within the Psychosocial Care Network (RAPS). Between 2017 and 2025, she worked in Psychosocial Care Centers (CAPS) and in specialized services for women experiencing violence, experiences that raised concerns about the persistence of gender inequalities in healthcare.

Especially during her time as a reference professional for the homeless population in the municipality of Campinas, the researcher developed prior knowledge about the dynamics of the Street Clinic's operation, its work processes, and the relationships established between professionals and users. Her departure from the professional field occurred in May 2025, ensuring a temporal distance before the start of data collection and helping to minimize possible influences resulting from the researcher's dual involvement in the field.

In the ethnographic context, this prior experience favored the process of insertion and acculturation in the field, allowing for greater familiarity with the codes, practices, languages, and forms of interaction shared by the participants. This condition facilitated access to interlocutors, the building of trusting relationships, and a contextualized understanding of the observed situations, contributing to a more qualified listening to the narratives produced during fieldwork. At the same time, it demanded a continuous exercise of reflexivity on the part of the researcher, seeking to identify and problematize the effects of her professional trajectory on observation, interaction with participants, and data interpretation, as advocated in ethnographic research (Berry 2005).

Researching women experiencing homelessness poses ethical challenges that extend beyond formal consent procedures. The vulnerability of these participants was not treated as an individual attribute, but as an effect of accumulated social and institutional inequalities throughout their lives. This required the researcher to pay constant attention to the power asymmetries inherent in the research relationship. Faced with narratives of violence and affective bonds, the decision was made not to insist on or delve deeper into accounts when there were signs of discomfort, prioritizing the well‐being of the participants over the completeness of the data. Furthermore, efforts were made to ensure that the decision to participate or refuse would not imply any alteration in the care offered by the Street Clinic, explicitly dissociating the research from the assistance received—care that is especially relevant in a population whose relationship with public services is frequently marked by experiences of institutional control and surveillance.

### 2.5 Data Collection Instruments and Procedures

Data production involved participant observation, field diary entries, and semi‐structured interviews. The interviews explored the participants' life trajectories and their experiences accessing health services, with particular attention to affective bonds, social networks, and the meanings attributed to care practices, as shown in Table 1.

**TABLE 1:** Semi‐structured interview script developed for the present study and used with homeless women accessing the Psychosocial Care Network (RAPS). Campinas, São Paulo, Brazil, 2025.

Thematic axis	Guiding questions
1. Sociodemographic characterization and trajectory on the street	What is your age, race/color, and sexual orientation? How long have you been homeless? And how long have you been in Campinas?
2. Perceptions about health and personal needs	What are your main health needs today? Do you feel that these needs are, or have been, met at some point?
3. Experiences with health services and the Psychosocial Care Network (RAPS)	Do you usually seek any health services? Which ones? Have you ever been seen by a CAPS (Psychosocial Care Center), Street Clinic, UBS (Basic Health Unit), SAMU (Mobile Emergency Care Service), or other RAPS (Psychosocial Care Network) service? What was that experience like? Have you ever been hospitalized? What was that experience like?
4. Barriers and difficulties in accessing services	Have you ever tried to seek help and been unable to? What difficulties do you face in accessing health services? Do you believe that being a homeless woman interferes with this access?
5. Perceptions about the quality of care	Do you notice a lack of resources in the services? Has this affected you in any way?
6. Suggestions and perspectives for care	What do you think could improve care for other women in the same situation? What would make it easier to access healthcare services?
7. Closing	Is there anything else you'd like to share that wasn't asked during the interview?
Additional question (when relevant)	Have you ever experienced violence? If you feel comfortable, could you share how that experience impacted your life and your search for healthcare?

*Source*: Research data.

Data collection was carried out until theoretical saturation was reached, understood as the point at which the information obtained began to present recurrence and redundancy, without the addition of new elements relevant to understanding the phenomenon investigated, as proposed by Fontanella et al. (2011).

### 2.6 Data Analysis Strategy

The collected material comprised the corpus analyzed via Thematic Content Analysis (Bardin 2011; Bernard et al. 2010). After the complete transcription of the interviews and the completion of a preliminary reading, the data were subjected to a systematic coding process into units of meaning. Based on the identification of regularities, convergences, and particularities present in the narratives, analytical categories were constructed that gave rise to the central themes of the study, privileging, whenever pertinent, emic expressions and native categories mobilized by the participants themselves. The elaboration of a codebook (a systematized framework of codes and analytical categories) and the continuous discussion among the researchers ensured the consistency and reproducibility of the analysis, culminating in the thematic axes that structure this article (Macqueen et al. 1998).

The data will be presented in the body of the text with the interlocutors' statements highlighted only in italics, without indentation or other graphic resources that establish hierarchies between their narratives and the researcher's writing. This choice is based on the perspective of Viveiros de Castro (2002), who questions the distinction between the researcher as the sole producer of theory and the interlocutors as mere informants. Thus, the participants in this research are understood as subjects who produce knowledge, elaborate interpretations about the world, and attribute meaning to their experiences. The use of italics, therefore, only serves to identify the different voices present in the text, keeping them on the same analytical plane and recognizing their contribution to the construction of the knowledge produced in this investigation.

The names used throughout this work are fictitious, in order to preserve the anonymity and confidentiality of the research participants.

### 2.7 Theoretical Framework

The analysis was grounded in the contributions of Zanello (2018) on mental health, gender, and culture, which enabled the analysis of gender intersections in women's subjectivation processes. Furthermore, the work of Carla Akotirene (2019) allowed for a deeper understanding of intersectionality as an analytical tool to examine the articulation between different social markers of difference and inequality. Complementarily, the work of Lélia Gonzalez (2020) provided an analysis that considered the intersectionality between race, gender, and class, highlighting how multiple oppressions are articulated in the participants' experiences.

In this way, the data analysis sought to articulate microsocial and macrosocial dimensions, understanding healthcare as a contextualized practice, traversed by power relations and structural inequalities. Thus, the analytical process made it possible to describe the observed practices, as well as to problematize the social determinants involved and their effects on access to care.

## 3 Results and Discussion

The participants' profiles are presented in Table 2. The study included 11 women aged between 27 and 56 years. The majority were brown and black women, heterosexual and cisgender, with the participation also of one transgender woman, one homosexual woman, and one bisexual woman.

**TABLE 2:** Characterization of the participants.

Fictitious Name	Gender identity	Race	Age	Sexuality
Rita	Cis woman	Black	49 years old	Heterosexual
Carolina	Cis woman	White	40 years	Homosexual
Nise	Cis woman	Brown	35 years	Heterosexual
Clarice	Cis woman	White	42 years old	Heterosexual
Conceição	Cis woman	Brown	42 years old	Heterosexual
Dandara	Cis woman	Brown	33 years old	Heterosexual
Elza	Cis woman	Black	45 years old	Heterosexual
Marielle	Cis woman	Black	27 years old	Bisexual
Madalena	Trans woman	White	43 years old	Heterosexual
Clementina	Cis woman	Brown	46 years old	Heterosexual
Maria	Cis woman	Brown	56 years old	Heterosexual

*Source*: Research data.

Based on the analysis of data produced in interviews, participant observation, and field diary entries, two thematic axes were identified. The first, entitled **“I lived with him, but he tried to kill me,”** brings together narratives about experiences of violence committed by current and former intimate partners, highlighting how these aggressions permeate the participants' trajectories, influence their entry into and persistence in homelessness, and impact their healthcare pathways. The second axis, **“I've dreamed of a white dress since I was a child,”** highlights the ambiguities of the affective bonds established with intimate partners.

“I lived with him, but he tried to kill me.”

Rita has been living on the streets for two years, ever since she left her hometown due to violence perpetrated by her ex‐husband: “ *I lived with him, but he tried to kill me, he tried to set me on fire*.” Upon arriving in the city, she sought refuge in a night shelter. However, as time passed, she began to move around other areas of the city. She mentions that, due to this situation, she lost contact with her children: *“My father took my children away.”*

Clarice has been homeless for about 8 months, also due to conflicts with her partner: “ *Living together is impossible, he's a very aggressive person,”* she says, with anguish, about the consequences of this violence, which led to other intra‐family conflicts and the non‐acceptance of the relationship by her children.

Dandara, in turn, reports that she frequently uses alcohol: *“I stopped drinking for a while, but I ended up going back because my husband cheated on me and I was very frustrated*.” Her statement reveals that the reasons that led her to the streets 18 months ago involve multiple factors, including the desire to be close to her partner. However, it is also a relationship marked by gender‐based violence: *“ The relationship with my husband is sometimes good, sometimes it isn't… Most of the time it isn't, he gets aggressive.”* The complexity of Dandara's situation became evident when, at the end of the interview, her partner passed by and told her: *“Don't even talk to me because I hate you.”*

Maria explains that living on the streets was the way she found to survive the serious violence committed by a former intimate partner: *“I lived with him 5 years ago, but he still stalks me, he doesn't accept anyone with me.”* She also mentions that, after the separation, he would break into her house: *“He would enter the house through the door, through the window, even through the roof tiles*.”

Clementina's case also involves brutal violence. During the interview, she points to a deep scar on her forehead and says, “ *Look, my ex bit me here. It hurt.”* Separated for two months, she recounts, distressed, the affection she had for her partner: *“I liked him, he wasn't always bad, but sometimes he used drugs and became like this, very aggressive. He even fractured my foot and dislocated my jaw.”*

The narratives presented demonstrate that the aggressions they suffered do not correspond to individual or isolated events, but rather relate to complex social dynamics, sustained by historically unequal relations between men and women. Understanding these experiences requires situating them within the context of social gender constructions that organize ways of living, loving, and exercising power in affective relationships (Zanello 2018).

In a patriarchal capitalist culture, there are many physical and psychological demands that function as true social prescriptions for the most honorable ways of existing. The acquisition of masculine attributes is characterized by a violent process, with violence being fundamental to masculinity itself. In contrast, femininity is socially constructed based on the valorization of care and affective availability (Zanello 2018).

This establishes a relationship of political, economic, and patriarchal domination by men, giving rise to what we call gender: a historical‐cultural construction of the attributes of masculinity and femininity marked by social expectations, anchored in a hierarchy and a dualistic vision that throughout history has defined men and women as radically opposed beings. Gender violence is then defined as that which occurs in a relationship based on asymmetry between genders (Zuma et al., 2007). Teles (2006) points out that it is from these unequal relationships that the debate on gender begins to be configured as “a political concept, according to which power, decisions, and privileges are in the hands of men” (p. 47), sustaining the maintenance of women as secondary subjects, for use and domination.

In this sense, hegemonic ideals of femininity have been historically constructed from white, bourgeois, and heteronormative references, establishing expectations about what a “good woman” should be. In this model, femininity is strongly associated with motherhood, caregiving, and domesticity, so that the social role attributed to women is defined primarily by the responsibility of caring for children and the need to own and look after a home (Zanello 2018; Gonzalez 2020).

Thus, Saffioti (2015) presents the imbrication between gender and social class from the perspective of the class society in which we live, where these differences and variations occur through social hierarchy, based on patriarchy, sexism, social class, and the issue of race/ethnicity. Building on this discussion, Arruzza, Bhattacharya, and Fraser (2019) expand this debate by presenting the alliance between patriarchy and capitalism, terming it patriarchalism, in which they point out how capitalism has organized forms of violence and oppression within its system, sustaining and solidifying patriarchy, intertwining this way of establishing social relations in our culture and social organization. Thus, the authors (Teles 2006; Saffioti 2015; Arruzza, Bhattacharya and Fraser, 2019) emphasize that the central element in this hierarchization of genders occurs through capitalism, in which what differentiates men from women is related to the essential role that each plays in the social reproduction of life and in maintaining the structure of this system.

When a woman experiences homelessness, it is understood that she “loses her home” and thus no longer has a space where she can perform the socially expected domesticity. In this context, she distances herself from the hegemonic ideal of femininity and comes to be perceived not only as a person experiencing homelessness, but also as a woman who has failed to fulfill the socially assigned roles of mother and homemaker.

This phenomenon is clearly observed in the accounts of Rita and Clarice. By losing their formal housing, they also lost direct contact with their children, suffering a double penalty: the violence perpetrated by their intimate partners and the deprivation of their symbolic role as mothers. Thus, gender norms operate in a way that blames them. This double penalty is not limited to the symbolic sphere: by losing contact with their children and their formal housing, Rita and Clarice also report greater difficulty in maintaining regular links with health services, since the territorial instability resulting from the marital breakdown fragments the longitudinal follow‐up offered by the Psychosocial Care Network (RAPS), demonstrating that gender violence operates as a direct determinant of the discontinuity of care.

However, gender does not operate in isolation in the production of inequalities and violence. As intersectional studies point out, women's experiences are simultaneously traversed by different social markers, such as race, class, territory, and housing conditions, which produce specific forms of oppression and vulnerability (Akotirene 2019). Thus, the violence experienced by the participants cannot be understood solely from their condition as women, but also from the social position they occupy as women, Black women, homeless women, and, in some cases, as those who use psychoactive substances. This is a complex dynamic of gender violence and socio‐racial segregation (Malheiro 2019).

Thus, these women are often perceived as transgressors of the normative models set by the ideal of femininity, and end up occupying a place of less social recognition. As a consequence, their demands tend to be delegitimized, which directly impacts access to public policies (Malheiro 2019).

From this perspective, the narratives reproduced here reveal that these women, seeking to break free from gender‐based violence, continue to be subjected to it and its multiple expressions, perpetrated by intimate partners and former partners, as well as by state agents (Richwin; Zanello 2023; Malheiro 2019).

Thus, violence also manifests itself in the institutions that should ensure protection and guarantee rights, as Maria reported: *“I went to the women's police station, but they didn't even give me a protective order (…) if you went to the police station to file a police report it would be different. Everywhere you go, we are discriminated against in society.”*

By establishing this comparison with the researcher, Maria highlights her perception that the care she received was influenced by social markers such as race, class, and housing condition, recognizing that a white, domiciled woman would be more easily legitimized. This is an intersectional analysis of her own condition. She acknowledges that access to rights and justice is not universal, but rather a privilege associated with skin color, social class, and address (Akotirene 2019).

Despite normative advances and institutional recognition of gender‐based violence as a public health issue, the health sector is not immune to the patriarchal structures that sustain this violence. On the contrary, such structures frequently permeate care practices, influencing how women are welcomed, listened to, and cared for. Thus, the formal recognition of gender inequality does not, in itself, guarantee the transformation of power relations present in the daily routine of services, which may continue to reproduce, subtly or explicitly, gender‐based violence and institutional violence (Minayo 2007; Villela et al. 2011).

Marielle is 27 years old, but has been homeless since childhood, more specifically since she was 10 years old, due to family conflicts. She mentions that, in her daily life, she usually accesses different health services, and recounts, with shame, an episode in which she was a victim of sexual violence committed by a doctor during a hospital stay (Extracted from Field Diary, September 30, 2025). In this case, a multiple power asymmetry is evident, involving medical knowledge‐power and the domination of the male gender in relation to the female gender acting on a body marked by the vulnerability of homelessness (Gonzalez 2020).

The impact of this trauma can lead to various consequences, including the discontinuation of care and the exclusion of these women from the formal health network (D'oliveira et al. 2002). This highlights a paradox in the social assistance and protection network, which fails to fulfill its fundamental role by leaving these women helpless, unprotected, and even subjected to violence (Richwin; Zanello 2023).

Finally, the narratives presented allow us to understand that gender‐based violence is a constitutive element in the trajectories of women experiencing homelessness: present in affective and institutional relationships, it permeates the processes that led to homelessness, operates in daily life producing the precariousness of care, including the actions and omissions of public authorities.

From this, many women begin to build their own survival and safety strategies which, as Richwin and Zanello (2023) point out, often involve intimate relationships in which they are again exposed to situations of violence, which have repercussions on the possibilities of access, permanence and building bonds with health services, as will be discussed below.

“I've dreamed of a white dress since I was a child.”

One issue that emerged during the interviews was the complexity of heterosexual romantic relationships. Of the 11 women interviewed, 8 attended the Street Clinic accompanied by a man, identified as a partner or friend. Although these companions did not participate in the interviews, which were conducted individually, their presence highlighted the relevance of these relationships in the participants' daily lives. The women described how these bonds, while offering affection and protection, can also become obstacles to accessing health services and leaving homelessness.

One can cite the example of Conceição, who was accompanied by a friend wearing a t‐shirt with the phrase *“I never made friends drinking milk”*. Ironically, they were both sitting in the square sharing bread and milk. Furthermore, Conceição reported that it was this friend who encouraged her to seek help at the Street Clinic (Extracted from Field Diary, September 12, 2025).

The scene is interesting because the phrase printed on the t‐shirt, commonly associated with alcohol consumption, appears at the same time as the two share food and a gesture of mutual care. This situation suggests that the bonds built in the territory involve forms of peer care.

Nise, for her part, is pregnant and sought out the Street Clinic to receive prenatal care and address issues related to substance abuse. She states: *“Sometimes I want to come to the clinic, sometimes I don't.” “I came today because my husband insisted*,” thus highlighting the role that community ties can play in accessing health services.

Rita, in turn, is in a relationship with a man who is also homeless. Speaking about the time he was imprisoned, she recounts: *“While he was in prison, it was very difficult for me.”* This narrative also indicates the importance of the marital bond in her daily life.

However, these relationships cannot be understood in a homogeneous or idealized way. The same relationships that offer companionship, protection, and encouragement for access to health services can also be permeated by different forms of gender‐based violence. In this sense, the participants' narratives reveal the ambivalence of these bonds.

In this sense, Clarice explains the reasons that keep her away from her home: “I even have a house, but he doesn't. And then I tried to get him to live with me, but he has a very strong temper, it didn't work. He doesn't have a house. Since my children don't accept it, to stay with him I need to stay here.” Her account highlights how gender‐based violence produces family ruptures and limits the possibility of remaining in spaces considered safe, contributing to the perpetuation of homelessness. Furthermore, it indicates that remaining on the streets may be related to various factors, including the importance of romantic relationships in her life.

Dandara recounts: “I even have a house, but I prefer to stay on the street because of my husband. I have a small farm with my family.” She then adds: “I can only go where he goes. I always want to take him with me. ”

It is worth noting that, in the ideal of femininity produced by the hegemonic heterosexual matrix, women are expected to be linked to a partner. As Malheiro (2019) points out, the constitution of women as subjects is historically associated with the presence of a partner, which contributes to the permanence of affective relationships even when these are marked by situations of violence.

Furthermore, it's important to highlight that Dandara's emotional state also directly interferes with her healthcare. She mentions that she and her partner were diagnosed with syphilis approximately six months ago and that she is facing difficulties completing the treatment: *“I can't do the treatment. I've already had the injection twice, but then I don't take it anymore.”* According to her, her partner's resistance makes it difficult to continue the care: *“What makes it difficult is the relationship because he doesn't want to do the treatment*.” In the end, she expresses concern about the situation: *“He doesn't want to. It's difficult, very difficult.”*

Dandara's narrative indicates that romantic relationships can directly influence care pathways and women's relationship with health services. While these relationships can facilitate access to and engagement with the Psychosocial Care Network (RAPS), they can also hinder access to care, treatments, continuity of follow‐up, and the use of available resources within the network. In this case, the partner's refusal to undergo treatment directly impacts the participant's care possibilities, demonstrating that access to services depends not only on institutional availability but also on the relational dynamics that permeate these women's daily lives.

In this sense, Richwin and Zanello (2023) describe the functioning of the “violence‐protection paradox,” according to which, in the context of life on the streets, many women remain in violent relationships as a survival strategy: the presence of a partner, even if the perpetrator of violence, can represent a form of protection against other, even more serious aggressions. Dandara's case illustrates a mechanism described in the international literature on intimate partner violence as partner interference with health care, a process by which the partner acts, directly or indirectly, hindering the woman's access to, adherence to, or continuation of treatment, either through explicit control or by maintaining dynamics of affective and financial dependence (McCloskey et al. 2007). In her account, this mechanism manifests itself in her partner's refusal to treat her syphilis, which compromises not only her adherence to treatment but also perpetuates a cycle of reinfection and shared illness, revealing how gender violence is materially inscribed on the body through the therapeutic trajectory itself.

Dandara's speech is also filled with romantic idealizations built since childhood: “I've dreamed of a white dress since I was a child… I dreamed of that, I dreamed of a wedding, I dreamed of a child after the wedding, I truly dreamed of great love.” Emotionally, she describes the recurring image of an idealized wedding: “I dreamed of a man and me in a very proper, white, beautiful dress.”

This account requires recognizing that the process of women's subjectivation is traversed by what Zanello (2018) calls the amorous apparatus. This is a set of social norms, values, and expectations that teach women that their personal fulfillment, social recognition, and subjective value depend on the establishment and maintenance of a loving relationship. As a consequence, affective relationships come to occupy a central place in their lives, constituting one of the main focuses of affective investment. In this context, understanding the importance attributed to marital bonds by the participants implies recognizing that such relationships are not only individual choices, but also products of social and cultural gender processes (Zanello 2018).

Considering the processes of social exclusion, these ideals can also be understood as attempts to reclaim subjective dignity through expectations of femininity anchored in gender norms. In this sense, marriage and conjugal life are integral to the construction of a “legitimate feminine identity,” from which these women have been distanced, as well as the possibility of transforming their lived material reality. It is observed, therefore, that intimate partner violence and institutional violence, although distinct in their origin, converge in the same effects on the therapeutic journey of these women. In the search for care, the fear of judgment and shame, visible in the constrained way in which Marielle recounted the abuse suffered, act as a barrier to entry into services. In access, the dependence on third parties to attend appointments, as in Nise's case, shows that arrival at the service does not always result from full autonomy. Regarding permanence and experience within the system itself, the institutional discredit reported by Maria and the violation suffered by Marielle within a health facility show that violence can be reproduced within the very devices that should offer protection. These three moments—search, access, and permanence—reveal that gender‐based violence does not act merely as a biographical backdrop, but as an active force that structures, at each stage, these women's relationship with healthcare.

In contrast, Elza brought an important perspective by expressing the importance of the bonds built with the professionals at the Street Clinic and other services within the Psychosocial Care Network (RAPS). She emotionally described how realizing she could be loved and cared for by other people, beyond a romantic partner, was transformative: *“When I realized that I can be loved, including by the service workers, I stopped being so attached to this idea that I have to be loved by a man.”*

Her statement points to the construction of support networks, which do not necessarily involve a male figure, demonstrating that when the health service adopts qualified listening and a welcoming posture, so as not to stigmatize the subject, it can reposition that user's place in the world and their relationship with care (Campos 2003).

Elza's account suggests that devices like the Street Clinic can act as a space for re‐signifying affective bonds, offering a care experience not conditioned on the mediation of a partner. This finding points to concrete paths for the development of the Psychosocial Care Network (RAPS) in the face of the challenges identified in this study: (1) the incorporation of systematic screening for intimate partner violence into the routine of the Street Clinic, and not only through spontaneous disclosure; (2) the formal and continuous articulation between the RAPS, the women's protection network, and social assistance in the face of reports of institutional violence, in order to prevent users from circulating between services without an effective response, as reported by Maria; and (3) the ongoing training of teams to recognize that adherence to treatment may depend on marital dynamics, as in the case of Dandara, which demands approaches focused on the couple or harm reduction strategies, instead of individual user responsibility.

## 4 Limitations of the Study

This study presents some limitations that should be considered when interpreting the results. Because it is a qualitative, ethnographically inspired study conducted in a single municipality with a small number of participants, the findings are not intended for statistical generalization, but rather for an in‐depth analytical exploration of the experiences investigated. The participants were recruited from among users of the Street Clinic, which may have favored the inclusion of women who already had some connection with health services. Therefore, it is suggested that future investigations seek to reach women who are outside the formal healthcare service circuit, expanding the mapping of access barriers and the most severe forms of institutional invisibility. Furthermore, the results reflect a specific time frame, corresponding to the period of fieldwork, and may not capture subsequent changes in the participants' trajectories, the affective bonds established, or the dynamics of access to health services. Despite these limitations, the combination of participant observation, semi‐structured interviews, and field diary entries enabled a deeper understanding of the effects of gender‐based violence and affective bonds on healthcare among homeless women.

## 5 Conclusion

Gender‐based violence has emerged as a central dimension in the trajectories of women experiencing homelessness, permeating the processes that led to the breakdown of relationships, the experience of street life, and their interactions with health services. Affective‐romantic bonds have been marked by a paradox between protection and vulnerability: while offering emotional support, belonging, and survival strategies, they can also restrict autonomy, hinder access to healthcare, and reproduce dynamics of violence. By situating this violence within its concrete interface with care pathways, and not merely as a biographical backdrop, this study contributes to filling a gap in the understanding of how gender‐based violence, both intimate and institutional, translates into specific barriers and possibilities for access, adherence, and retention in the services of the Psychosocial Care Network.

In this sense, it becomes fundamental that the services of the Psychosocial Care Network (RAPS) and other public policies develop strategies capable of recognizing the impacts of gender relations on the production of care, expanding spaces for listening, welcoming, and building bonds. Finally, it is hoped that this study will broaden the debate on the specificities of healthcare for women experiencing homelessness, strengthening practices and policies committed to equity, guaranteeing rights, and confronting the multiple forms of violence that permeate their lives.

## Funding

The Article Processing Charge for the publication of this research was funded by the Coordenação de Aperfeiçoamento de Pessoal de Nível Superior ‐ Brasil (CAPES) (ROR identifier: 00x0ma614).

## Ethics Statement

The study was conducted in accordance with Resolutions No. 466/2012 and No. 510/2016 of the Brazilian National Health Council and was approved by the Institutional Research Ethics Committee (CAAE No. 90556725.7.0000.5418).

## Patient Consent

The Participation was formalized through the signing of the Informed Consent Form, ensuring anonymity, information confidentiality, and the autonomy of the participants.

## Conflicts of Interest

The authors declare that there are no financial or personal conflicts of interest that could have influenced the content of this article.

## Data Availability

The qualitative data generated in this study (interview transcripts and field diaries) are not publicly available to ensure the anonymity and protection of the participants, in accordance with approved ethical guidelines. The data remain under the responsibility of the corresponding author.
